# Efficacy of dietary vitamin D_3_ and 25(OH)D_3_ on reproductive capacities, growth performance, immunity and bone development in pigs

**DOI:** 10.1017/S0007114523000442

**Published:** 2023-10-28

**Authors:** Maruf Hasan, Michael Oster, Henry Reyer, Klaus Wimmers, Dagmar-Christiane Fischer

**Affiliations:** 1 Research Institute for Farm Animal Biology (FBN), Wilhelm-Stahl-Allee 2, 18196 Dummerstorf, Germany; 2 Department of Pediatrics, Rostock University Hospital, Ernst-Heydemann-Str. 8, 18057 Rostock, Germany; 3 Faculty of Agricultural and Environmental Sciences, Justus-von-Liebig-Weg 6b, University of Rostock, 18059 Rostock, Germany

**Keywords:** Calcitriol, Farm animals, Mineral homeostasis, Nutritional programming

## Abstract

Vitamin D_3_ (Vit D_3_) and 25(OH)D_3_ are used as dietary sources of active vitamin D (1,25(OH)_2_D_3_) in pig husbandry. Although acting primarily on intestine, kidney and bone, their use in pig nutrition has shown a wide range of effects also in peripheral tissues. However, there is an ambiguity in the existing literature about whether the effects of Vit D_3_ and 25(OH)D_3_ differ in attributing the molecular and phenotypic outcomes in pigs. We searched Web of Science and PubMed databases concerning the efficacy of Vit D_3_ in comparison with 25(OH)D_3_ on pig physiology, i.e. reproductive capacities, growth performance, immunity and bone development. Dietary intake of Vit D_3_ or 25(OH)D_3_ did not influence the reproductive capacity of sows. Unlike Vit D_3_, the maternal intake of 25(OH)D_3_ significantly improved the growth performance of piglets, which might be attributed to maternally induced micronutrient efficiency. Consequently, even in the absence of maternal vitamin D supplementation, 25(OH)D_3_-fed offspring also demonstrated better growth than the offspring received Vit D_3_. Moreover, a similar superior impact of 25(OH)D_3_ was seen with respect to serum markers of innate and humoral immunity. Last but not least, supplements containing 25(OH)D_3_ were found to be more effective than Vit D_3_ to improve bone mineralisation and formation, especially in pigs receiving basal diets low in Ca and phosphorus. The insights are of particular value in determining the principal dietary source of vitamin D to achieve its optimum utilisation efficiency, nutritional benefits and therapeutic potency and to further improve animal welfare across different management types.

## Graphical abstract

**Figure:**
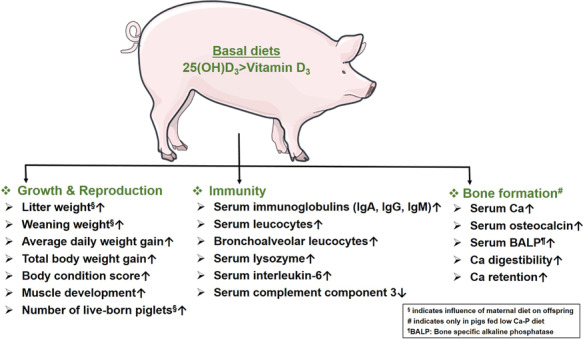

Vitamin D_3_, cholecalciferol (Vit D_3_) and 25-hydroxycholecalciferol (25(OH)D_3_) are the two major dietary forms to supply the organism with vitamin D (Vit D). In recent years, 25(OH)D_3_ is being studied as an alternative to Vit D_3_, as it is more bioavailable, efficiently absorbed, bypasses hepatic metabolism and is three to five times more potent than Vit D_3_^(1)^. Nevertheless, both dietary forms of Vit D are biologically inactive and require two (Vit D_3_) and one sequential hydroxylation reaction (25(OH)D_3_) for activation (Fig. 1). After ingestion or dermal synthesis, Vit D_3_ is transported via Vit D-binding protein (DBP) and hydroxylated via Vit D 25-hydroxylase (encoded by *CYP2R1*) in liver to form 25(OH)D_3_ and via 25-hydroxyvitamin D 1-alpha-hydroxylase (encoded by *CYP27B1*) in kidney to 1,25(OH)_2_D_3_ (the active form of Vit D). The excess amount of 1,25(OH)_2_D_3_ is subject to renal elimination following CYP24A1-mediated hydroxylation^(2)^. Nevertheless, both synthesis and elimination also occur in non-renal tissues (i.e. intestine)^(3)^. After activation, 1,25(OH)_2_D_3_ migrates via DBP to various target tissues such as intestine, bone, muscle, immune system, kidney, parathyroid glands and reproductive system to attribute its functions.

**Fig. 1.**
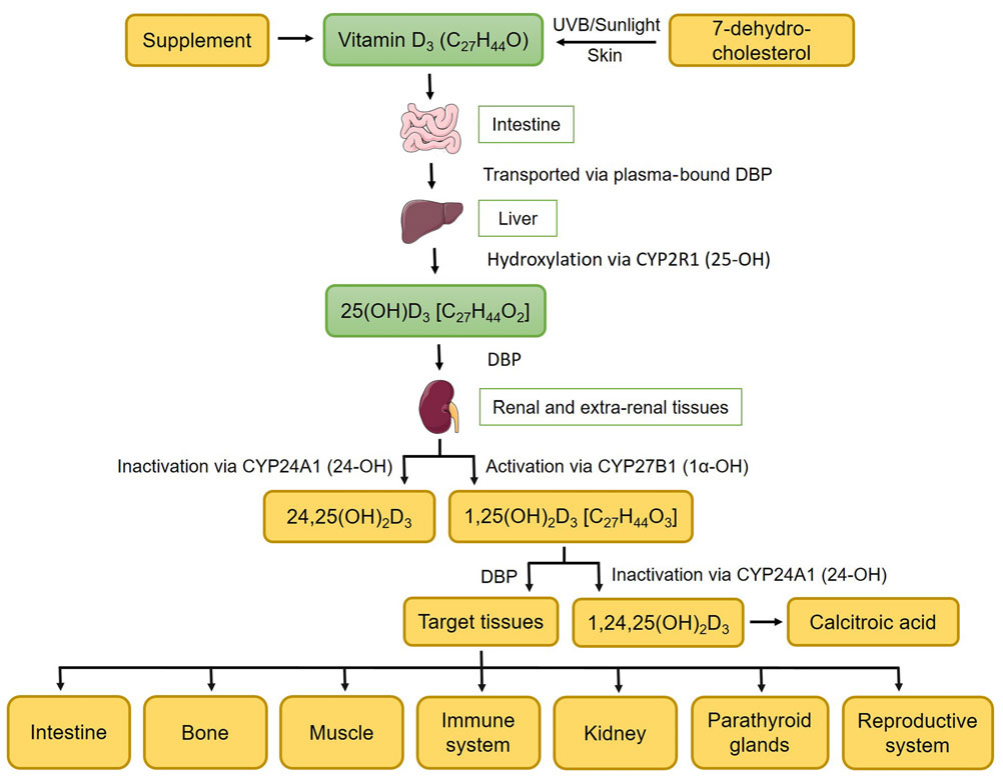
Schematic illustration of the endogenous production of active (1,25(OH)_2_D_3_) and inactive (1,24,25(OH)_2_D_3_) forms of vitamin D from supplemented or dermally synthesised vitamin D_3_.

The mediation of the biological functions of Vit D depends on its successful binding with the intracellular Vit D receptor (VDR, ligand-dependent transcription regulatory molecule) forming the VDR-1,25(OH)_2_D_3_ complex. This complex interaction initiates the formation of two autonomous protein interaction surfaces on the VDR; one of them modulates interplay with the retinoid X receptor (RXR) for DNA binding and the other recruits co-regulators to control gene expression. After dimerisation of VDR-1,25(OH)_2_D_3_ with RXR, the heterodimer translocates to the nucleus, binds to the Vit D responsive element (VDRE), which in turn regulates the expression of numerous genes (> 900), and implements the specific function of Vit D in particular tissues^(4,5)^.

The secosteroid hormone Vit D is critical for the maintenance of serum Ca and P homeostasis for optimum bone development, as it modulates the active uptake of minerals through the intestine^(6,7)^. The tissue-wide expression of VDR and other genes involving Vit D metabolism underscores the fact that the function of Vit D is not limited to osteogenesis or serum mineral balance^(3,8)^. In fact, an array of tissues are capable to express relevant genes encoding hydroxylation enzymes for calcitriol synthesis and elimination in mammals^(3,9)^. Thus, it turns out to be equally important for growth^(10–13)^, immunity^(14,15)^, oxidative status^(16,17)^, reproductive capacity^(18,19)^ and progeny performance^(20)^. In pig feeding, legislation limits the Vit D supply to 1000–2000 μg/kg diet, which corresponds to 25–50 µg/kg feed^(21,22)^, while 25(OH)D_3_ in combination with Vit D_3_ is allowed up to doses of 50 µg/kg feed^(23)^. However, the most potent form of Vit D in terms of nutritional benefits remains unclear, largely due to divergent functional demands of tissues and cell types^(3,9)^. Given the background, this review summarises the available literature for molecular and phenotypic outcomes of Vit D_3_ in comparison with 25(OH)D_3_ supplements in terms of reproductive capacities, growth performance, immunity and bone development in pigs.

## Search strategies and selection of articles

We conducted a systematic query in Web of Science and PubMed to retrieve all the articles dealing with Vit D_3_ and 25(OH)D_3_ in pigs published from 1 January 2000 to 23 May 2022. The search strategy included appropriate MeSH terms (supplementary material S1) without any language restriction. In total, 454 articles from the Web of Science and 241 articles from PubMed were identified using the appropriate search query. Of these, a total of thirty-five articles were selected for review, paying attention to relevance, overlap and experimental design (supplementary material S2).

## Comparative performance of vitamin D_3_ and 25(OH)D_3_ on reproduction and growth

Recent investigations suggest that the dietary supply of both Vit D_3_ and 25(OH)D_3_ plays an important role in fertility and maturation. The objective of this section was to identify arguments for the more effective form of Vit D in the diet to improve sow reproductive capacities and growth performance of the piglets (Table 1). Parts of the reported findings could be attributed to adaptive responses resulting from maternal nutritional programming, i.e. long-term consequences for growth, function and structure of various tissues and cell types, and thus for health and welfare^(24)^.

**Table 1.** Overview of the comparative studies between vitamin D_3_ (Vit D_3_) and 25(OH)D_3_ supplementation, indicating the study designs, dosages of supplements, underlying conditions and significant results of the experiments with regard to sow reproductive capacity and growth performance of their progeny. Only supplemented diets (control and treatment) and the conditions that led to noticeable outcomes are listed

Authors	*n*	DTP† (days)	Doses of vitamin D (µg/kg)		Conditions	Outcomes linked to reproduction and growth performance*	
			Vit D_3_ ‡	25(OH)D_3_		Vit D_3_ > 25(OH)D_3_	25(OH)D_3_ > Vit D_3_
Thayer *et al.*^(25)^	69 sows 216 piglets	ET^a^ (S^b^) 0–59 (P^c^)	37·5	37·5 (Vit D_3_) + 50	MNDE^d^	–	↑Primary muscle fibre number
Upadhaya *et al.*^(20)^	160 piglets 48 sows	1–140	50	50	PDE^e^	↑*MSTN*^g^	↑ADWG^h^ (days 1–42, 99–140, 1–140), ↑FE^i^ (days 1–42), ↑ADFI^j^ (days 1–140), ↑*MYF5*^k^
			50	50	MDE^f^	↑*MSTN*	↑ADWG, ↑FE (days 1–42); ↑*MYOD*^l^, ↑*MYF5*
Upadhaya *et al.*^(18)^	48 sows	–	50	50	–	–	↑Survivability, ↑Weaning weight, ↑ADWG (P); ↑Body condition score, ↑Body weight gain (S)
Weber *et al.*^(19)^	227 sows	ET	50	50	MDE	–	↑Total litter weight, ↑Birth weight
	39 sows	ET	50	50	MDE	↑Weight gain (P)	–
Witschi *et al.*^(29)^	39 sows	ET	50	50	MDE	–	–
Zhang *et al.*^(30)^	24 sows	ET	50	50	MDE	–	↑Litter weight, ↑Piglet weight (day 21 of lactation); ↑Total litter weight gain
Hines *et al.*^(12)^	40 sows	ET	62·5	12·5 (Vit D_3_)+ 50	MDE	–	↑Total muscle fibre number; ↑Pax7+ Myoblast (cell culture, 72 h and 96 h)
Zhang *et al.*^(10)^	144 piglets	14/28	62·5	50	–	–	↑ADWG (days 15–28, 1–28), ↑Body weight (day 28)
Zhao *et al.*^(31)^	240 piglets	–	50	50	–	–	↑ADWG (10–20 kg)

^a^ET, explained on text; ^b^S, sows; ^c^P, piglets; ^d^MNDE, maternal-nursery diet effect on piglets; ^e^PDE, piglet diet effect; ^f^MDE, maternal diet effect on piglets; ^g^ *MSTN*, myostatin; ^h^ADWG, average daily weight gain; ^i^FE, feed efficiency; ^j^ADFI, average daily feed intake; ^k^ *MYF5*, myogenic factor 5; ^l^ *MYOD*, myogenic differentiation.

* Asterisk indicates significant outcomes (*P* < 0·05).

† DTP, dietary treatment period.

‡ 1 µg = 40 μg Vit D_3_.

Thayer *et al.*^(25)^ studied the effects of feeding Vit D_3_ and 25(OH)D_3_ on sow reproductive capacity, muscle fibre morphometry and subsequent growth performance of piglets. For this purpose, sixty-nine sows were randomly allocated to one of three dietary groups comprising (i) Vit D_3_ as control (37·5 µg/kg), (ii) Vit D_3_ and 25(OH)D_3_ at low levels (DL, 12·5 µg/kg Vit D_3_ + 25 µg/kg 25(OH)D_3_) or (iii) Vit D_3_ and 25(OH)D_3_ at high levels (DH, 37·5 µg/kg Vit D_3_ + 50 µg/kg 25(OH)D_3_). On the other hand, the piglets (*n* 216) from these sixty-nine sows were treated similarly to their mothers from birth until day 59 of life. No significant effect was observed on the reproductive performance of sows regardless of the dietary Vit D regimens. At the time of birth, piglets from DH-fed sows showed a significant increase in the number of primary muscle fibres in contrast to piglets from control sows at birth. However, this effect was no longer present when weaning piglets were investigated. During the nursery period, there was no effect of dietary Vit D sources on the growth performance, except for feed efficiency from day 28 to 59 and day 0 to 59. During this period, piglets from DH-fed sows showed a significant increase in feed efficiency, i.e., the body weight gain to feed intake ratio, in contrast to piglets from sows assigned to DL. Overall, dietary intake of 25(OH)D_3_ resulted in a significant increase in the number of primary muscle fibres at birth, but the total number of muscle fibres did not improve at birth or weaning.

Upadhaya *et al.*^(18,20)^ conducted two different studies to compare the effects of maternal Vit D_3_ and 25(OH)D_3_ supplementations on sow reproduction and offspring growth performance. In one study^(20)^, forty-eight multiparous sows were provided a basal diet containing Vit D_3_ (CON, 50 µg/kg Vit D_3_) or 25(OH)D_3_ (TRT, CON + 50 µg/kg 25(OH)D_3_) depending on their body weight and expected farrowing date. At weaning, eighty piglets each from CON and TRT sows were fed diets containing 62·5 µg/kg Vit D_3_ (weaning diet) and 43·75 µg/kg Vit D_3_ (growing-finishing diet) with or without 25(OH)D_3_ (50 µg/kg) for 140 d. Unlike Vit D_3_, the maternal intake of 25(OH)D_3_ significantly improved the average daily weight gain (ADWG) and feed efficiency of the piglets at the early stage of the nursery period. However, in the later phase of the nursery period, maternal ingestion of either source of Vit D demonstrated a similar effect on the growth performance of the piglets. In this period, the piglets alone supplemented with 25(OH)D_3_ showed significant improvement in ADWG, feed efficiency and average daily feed intake. Moreover, in contrast to Vit D_3_, 25(OH)D_3_ significantly increased the water-holding capacity and reduced pork drip loss. Feeding 25(OH)D_3_ to sows and their progenies had a significant impact on the expression of candidate genes associated with muscle formation. After dietary supplementation to sows and their offspring (post-weaning diet) with 25(OH)D_3_, the up-regulation of myogenic markers *MYOD1* (myogenic differentiation 1), *MYF5* (myogenic factor 5) and down-regulation of *MSTN* (myostatin) were observed, suggesting the importance of 25(OH)D_3_ for muscle development. MYOD1 and MYF5 are involved in the regulation of myoblast proliferation and differentiation and critically determine the survival of muscle progenitor cells as well^(26,27)^. On the contrary, MSTN is the negative regulator of muscle development^(28)^. In the second study^(18)^, a total of forty-eight multiparous sows received either a basal diet fortified with Vit D_3_ (control, 50 µg/kg) or a control diet containing 25(OH)D_3_ (TRT, 50 µg/kg) based on their body weight and expected farrowing date. The sows fed 25(OH)D_3_ demonstrated a significant increase in body weight gain and body condition score during the suckling period of the piglets. Unlike Vit D_3_, piglets from sows fed 25(OH)D_3_ also had significantly higher survival, ADWG and weaning weight than piglets from sows fed the control diet. These results indicate that dietary supplementation with 25(OH)D_3_ significantly enhanced growth performance in both sows and their offspring.

To compare the two dietary forms of Vit D in terms of reproductive performance, Weber *et al.*^(19)^ performed their study into two parts. First, 227 primi- and multiparous sows received a basal diet containing Vit D_3_ (114 sows, 50 µg/kg) and 25(OH)D_3_ (113 sows, 50 µg/kg) from mating to day 110 of gestation. 25(OH)D_3_-supplemented sows exhibited a significant positive impact on total litter weight and total weaning weight of the offspring compared with sows that received Vit D_3_. The intrauterine development of the embryos was also positively correlated with the maternal serum levels of 25(OH)D_3_. For the second experiment, thirty-nine sows received basal diets fortified with Vit D_3_ (DL, 5 µg/kg; DN, 50 µg/kg) and 25(OH)D_3_ (DH, 50 µg/kg) from the day of the mating and the treatments were continued for four reproductive cycles. Following the intake, the two dietary sources of Vit D exhibited similar impacts on sow reproductive performance. However, a significant increase in weight gain between birth and weaning was observed in the offspring of the DN-fed sows, in contrast to the offspring of DL- and DH-fed sows. In summary, the authors concluded that sows fed 25(OH)D_3_ showed a significant improvement in the birth weight of their offspring. But sows provided with either form of Vit D for more than one reproductive cycle may not show any noticeable impact on the growth performance of their progeny.

Witschi *et al.*^(29)^ conducted a comparative study in thirty-nine primi- and multiparous sows randomly assigned to Vit D_3_ (DL, 5 µg/kg or DN, 50 µg/kg) and 25(OH)D_3_ (HD, 50 µg/kg) from the day of mating to day 21 of lactation. Similar to other studies, the serum level of 25(OH)D_3_ increased significantly in pigs after the administration of 25(OH)D_3_ compared with Vit D_3_. DL led to a decrease in average daily feed intake and a tendency for a decrease in body weight and body weight gain compared with the other groups. Additional supplementation of HD had no significant effect. So, maternal diets supplemented with either form of Vit D (50 µg/kg) demonstrated a similar impact on the growth performance of their offspring.

Zhang *et al.*^(30)^ compared Vit D_3_ (50 µg/kg) and 25(OH)D_3_ (50 µg/kg) to determine their relative effects on the reproductive capacity of twenty-four sows and the growth performance of their offspring from day 107 of gestation to day 21 of lactation. No significant effect of Vit D treatments on sow reproductive performance was observed. However, in contrast to Vit D_3_, offspring from 25(OH)D_3_-fed sows showed a significant increase in litter weight, total litter weight and piglet weight gain during the lactation period. Unlike Vit D_3_, ADWG and total weight gain also tended to improve in piglets from 25(OH)D_3_-supplemented sows. Overall, the dietary supplementation of 25(OH)D_3_ in pregnant sows significantly improved piglet growth performance (5·3 % increase in weight gain on average).

Hines *et al.*^(12)^ studied the effects of maternal supplementation of Vit D_3_ or 25(OH)D_3_ on piglet growth and muscle development. They provided forty sows with a control diet containing Vit D_3_ (62·5 µg/kg) and an experimental diet fortified with 25(OH)D_3_ (DH, 12·5 µg/kg Vit D_3_ + 50 µg/kg 25(OH)D_3_) beginning 43 d before artificial insemination through day 90 of gestation. Both diets contained 12·5 µg/kg of Vit D_3_ to avoid possible Vit D deficiency. In comparison with Vit D_3_, foetuses from 25(OH)D_3_-fed dams showed a significant increase in the number of *longissimus dorsi* muscle fibres by 9·3 %. However, there were no significant effects of the maternal diet on the cross-sectional area of the muscles in piglets. Piglets from 25(OH)D_3_-fed sows had more Pax7+ myoblast (72 and 96 h of post-plating) in the *longissimus dorsi* muscle than piglets from sows fed Vit D_3._ Foetuses from 25(OH)D_3_-fed sows also had increased total myoblast proliferative capacity. Thus, a maternal diet containing 25(OH)D_3_ exhibited a significant positive impact on foetal muscle development.

In contrast to the aforementioned studies, there are very few studies that have investigated the growth performance after supplementing two dietary sources of Vit D directly to piglets rather than to pregnant sows. In the study of Zhang *et al.*^(10)^, 144 piglets were randomly assigned to three different dietary treatments with a follow-up period of 28 d, namely a normal Ca-P (PC), low Ca-P (NC) diet supplemented with 62·5 µg/kg Vit D_3_ and low Ca-P diet supplemented with 25(OH)D_3_ (NC + 25D, 50 µg/kg 25(OH)D_3_). The experiment was conducted for 28 d and consisted of phase 1 (days 0–14) and phase 2 (days 15–28). PC diet contained 0·81 % Ca, 0·60 % total P and 0·72 % Ca, 0·53 % total P, and NC diet contained 0·56 % Ca, 0·47 % total P, and 0·45 % Ca, 0·39 % total P in phase 1 and phase 2, respectively. Compared to NC, the body weight (day 28) and ADWG (days 15–28 and days 1–28) of the weaned piglets increased significantly after their dietary ingestion of NC + 25D. Overall, the piglets fed 25(OH)D_3_ showed noticeable growth performance even when no dietary source of Vit D was provided to their mothers.

Corresponding to the study of Zhang *et al.*, Zhao *et al.* also conducted feeding trials only in piglets to evaluate growth efficiencies^(31)^. They supplemented a total of 240 weaned piglets (21 d of age, initial body weight about 6 kg) with a positive control diet (PSC, 50 µg/kg Vit D_3_), negative control diet (NC, –0·15 % P and –0·25 % Ca of PSC), phytase (Phy diet, NC + 37·5 µg/kg phytase) and 25(OH)D_3_ (HyD, NC + 50 µg/kg 25(OH)D_3_; Phy + HyD, NC + 37·5 µg/kg Phy + 50 µg/kg HyD). The experiment was divided into two phases (6–10 kg and 10–20 kg). Regardless of the body weight, HyD supplements showed no significant effect in average daily feed intake or feed efficiency of the weaned piglets in contrast to NC. However, ADWG of piglets (10–20 kg) increased noticeably after dietary intake of HyD compared with NC. In essence, piglets from Vit D-deprived sows demonstrated noticeable improvement in their growth after dietary ingestion of 25(OH)D_3_ without altering their feed intake or feed efficiency. However, data on body composition traits have not been reported in this study.

Thus, most studies show that sows fed either form of Vit D have a similar impact on their reproductive ability. However, the survivability of piglets from sows fed 25(OH)D_3_ is significantly better than piglets from sows fed Vit D_3_^(32,33)^. Unlike Vit D_3_, feeding 25(OH)D_3_ to sows helps to noticeably increase offspring growth performance by improving weaning weight, ADWG, total body weight gain and body condition scores. In the absence of maternal supply of Vit D sources, supplementation of 25(OH)D_3_ to the offspring alone can also significantly boost their growth performance. 25(OH)D_3_ also outperforms Vit D_3_ in increasing the number of muscle fibres and improving the proliferation and differentiation ability of muscle cells.

## Comparative performance of vitamin D_3_ and 25(OH)D_3_ on immunity

In recent years, a great deal of research has been conducted to investigate the immunoregulatory functions of Vit D. This section aimed to discuss the efficacy of Vit D_3_ and 25(OH)D_3_ to promote immunity in pigs (Table 2).

**Table 2.** Overview of the comparative studies between vitamin D_3_ (Vit D_3_) and 25(OH)D_3_, demonstrating the study designs, dietary doses, underlying conditions and significant outputs of the experiments corresponding to immunity. Only the dietary doses (control and treatment) and the conditions that led to noticeable outcomes are listed

Authors	*n*	DTP† (days)	Doses of vitamin D (µg/kg)		Conditions	Outcomes linked to immunity*	
			Vit D_3_ ‡	25(OH)D_3_		Vit D_3_ > 25(OH)D_3_	25(OH)D_3_ > Vit D_3_
Zhang *et al.*^(10)^	144 piglets	1–28	62·5	50	Day 14 (low Ca-P)	↑C3^d^ (serum)	–
					Day 28 (low Ca-P)	–	↑IgG^f^, ↑IgA^g^, ↑IgM^h^, ↑CAT^i^ (serum)
					–	–	↑SCFA^j^ (feces)
Zhang *et al.*^(30)^	24 sows	ET^a^	50	50	–	–	↑*ACCA*^k^, ↑*FAS*^l^; ↑IgG (milk), ↑Butyrate (cecum)
Konowalchuk *et al.*^(15)^	149 piglets	14/21	50	50	Serum	–	↑TLC^m^, ↑GLC^n^, ↑LPC^o^ (day 14); ↑TPLC^p^
					BA^b^	–	↑PLC^q^ (day 21)
					Cell cultures	–	↑TMNC^r^, TLC, TBLC^s^ (viable; 24 h)
Upadhaya *et al.*^(20)^	48 sows	ET	50	50	–	–	–
	160 piglets	1–140	62·5	50	Day 42	↑IL-6^e^ (serum)	–
			43·75	50	Day 140	–	↑IL-1^t^ (serum)
Meuter *et al.*^(49)^	160 piglets	48	50	50	21 d (PW^c^)	–	↑IgG, ↑Lysozyme (serum)
					48 d (PW)	–	↑IgG, ↑Lysozyme (serum)

^a^ET, explained on text; ^b^BA, bronchoalveolar; ^c^PW, post-weaning; ^d^C3, complement component 3; ^e^IL-6, interleukin 6; ^f^IgG, immunoglobulin G; ^g^IgA, immunoglobulin A; ^h^IgM, immunoglobulin M; ^i^CAT, catalase; ^j^SCFA, short-chain fatty acids; ^k^ *ACCA, acetyl-CoA carboxylase α*; ^l^ *FAS*, *fatty-acid synthase*; ^m^TLC, total leucocyte count; ^n^GLC, granulocytes; ^o^LPC, lymphocytes; ^p^TPLC, total phagocytic leucocytes; ^q^PLC, phagocytic leucocytes; ^r^TMNC, total monocytes; ^s^TBLC, total bronchoalveolar leucocytes; ^t^IL-1, Interleukin 1.

* Asterisk indicates significant outcomes (*P* < 0·05).

† DTP, dietary treatment period.

‡ 1 µg = 40 μg Vit D_3_.

In the study of Zhang *et al.*^(10)^, 25(OH)D_3_ was more effective than Vit D_3_ in fortifying the body’s immune system when receiving a low Ca-P diet. In contrast to NC, dietary supplementation of 25(OH)D_3_ (NC + 25D) reduced the incidence of streptococcal infections by significantly increasing the serum concentrations of immunoglobulin A (IgA) and G (IgG) in piglets at weaning. Since these two types of immunoglobulins (Igs) are involved in inhibiting streptococcal and other bacterial infections^(34,35)^. Furthermore, in pigs receiving the 25(OH)D_3_ supplement, the serum concentration of IgM was enhanced at day 28, while one of the complement components (C3) was reduced at day 14. Compared to NC, diets containing 25(OH)D_3_ led to a significant rise in the serum concentration of catalase (CAT) at day 28, and this might be taken as a hint toward an improved antioxidant capacity. CAT is regarded as a first-line defence antioxidant enzyme that is fundamental and vital to the overall defensive mechanisms in biological systems^(36)^. However, NC and NC + 25D demonstrated similar effects on the serum status of other oxidative enzymes like super oxidase dismutase, total antioxidant capacity (T-AOC) and glutathione peroxidase (GSH-Px). In contrast to Vit D_3_, the intestinal concentration of SCFA significantly increased following the dietary intake of NC + 25D. For example, the abundance of Lachnospiraceae, which plays a crucial role in maintaining gut health, increased. The principal function of Lachnospiraceae is to break down the complex polysaccharide into SCFA, including acetate, butyrate and propionate^(37)^. SCFA are involved in boosting immunity and reducing inflammatory reactions in the gut and other organs by, i.e. acetyl-CoA synthesis, suppression of histone deacetylase, signalling via G protein-coupled receptors and metabolic integration^(38)^. Thus, dietary inclusion of 25(OH)D_3_ resulted in a significant improvement in humoral as well as innate immunity and gut immunity of the weaned piglets.

In another study, Zhang *et al.*^(30)^ compared the immune competence of sows supplemented with Vit D_3_ and 25(OH)D_3_. Sows fed either source of Vit D demonstrated a similar effect on the level of Igs (IgA, IgG and IgM) in colostrum. However, diet containing 25(OH)D_3_ significantly increased IgG levels in milk on day 21 of lactation. The expression of genes concerning fatty acid metabolisms, i.g., *ACCa* (Acetyl-CoA carboxylase *α*) and *FAS* (fatty-acid synthase) increased considerably in the mammary gland in response to 25(OH)D_3_ enriched diets. Lipid metabolism is directly involved in the regulation of the immune system through the activation of M1 and M2 macrophages^(39)^. The piglets from 25(OH)D_3_-fed sows demonstrated a significant elevation in the concentration of butyrate in the cecal digesta compared with piglets from sows fed Vit D_3_. The elevated concentration of butyrate following the breakdown of dietary fibres indicates improvement in bacterial metabolism^(40,41)^ which plays a crucial role in adaptive immune response via two specific pathways: first, it acts directly on monocyte-derived dendritic cells^(42–44)^ and second through its action on T lymphocytes. So, in contrast to Vit D_3_, the dietary inclusion of 25(OH)D_3_ during lactation period led to a significant increase in the concentration of milk IgG; and butyrate concentration in the cecal digesta of suckling piglets due to improved bacterial metabolism in the gut.

Konowalchuk *et al.*^(15)^ compared Vit D_3_ and 25(OH)D_3_ to evaluate the immune capacity by offering three different dietary treatments to 149 piglets weighing 5–7 kg for 14 or 21 d. The dietary supplements contained a baseline diet mixed with Vit D_3_ (NC, 37·5 µg/kg), which served as a control diet. The second regimen included the control diet mixed with an additional 50 µg/kg of Vit D_3_ (PC, NC + 50 µg/kg Vit D_3_). The third one consisted of the control diet mixed with 25(OH)D_3_ (HyD, NC + 50 µg/kg 25(OH)D_3_). Leucocytes are involved in both innate and humoral immune responses and play a critical role in fighting against infections and defence against foreign elements^(45)^. The authors observed that supplementation of 25(OH)D_3_ resulted in a significant increase in total blood leucocyte (granulocytes, lymphocytes) counts in piglets, accompanied by a parallel increase in serum 25(OH)D_3_. Compared with NC, piglets fed HyD demonstrated a significant improvement in the viability of total blood leukocytes and monocytes. The HyD-fed group also showed a significant increase in the viability of bronchoalveolar leukocytes compared with PC. However, unlike Vit D_3_, 25(OH)D_3_ did not only increase the number and viability of leucocytes but also significantly improved their phagocytic capacity. Overall, dietary supplementation of 25(OH)D_3_ compared with Vit D_3_ showed a significant positive influence on systemic blood and peripheral bronchoalveolar mucosal compartments, resulting in an increase in leukocyte count as well as the survival and phagocytic ability of the discrete leucocyte populations.

In the study of Upadhaya *et al.*^(20)^, considerably lower serum levels of interleukin-6 (IL-6) at day 42 and higher serum levels of interleukin-1 (IL-1) at day 140 were observed in growing pigs supplemented with 25(OH)D_3_ compared with Vit D_3_. IL-6 is an anti-inflammatory cytokine that contributes to host defence via stimulation of the acute phase response, haematopoiesis and immune responses^(46)^. IL-1 is a strong inflammatory cytokine involved in a wide range of immunological responses. It is predominantly produced by macrophages during defensive reactions to protect the body from infection and disease through inflammation and innate and adaptive immune responses^(47)^. The authors were unable to explain the underlying reason for this up- or down-regulation of the pro- and anti-inflammatory cytokines. According to Tanaka *et al.*, the fluctuation of inflammatory cytokines is due to the maintenance of immune homoeostasis^(48)^. In conclusion, the supplementation of 25(OH)D_3_ exhibited a more positive impact than Vit D_3_ on pig health.

Meuter *et al.*^(49)^ compared two different dietary sources of Vit D to observe their effects on humoral immunity by measuring the serum concentration of IgG and lysozyme. For this purpose, 160 post-weaned piglets (weaned at 21 days of age) were divided into two groups matched for maternal origin and body weight. Each group included sixteen pens with five animals each and received the same diet, differing only in the source and doses of Vit D used. The piglets received either Vit D_3_ (50 µg/kg) or 25(OH)D_3_ (50 µg/kg) for 48 d. The serum concentration of 25(OH)D_3_ increased considerably after dietary supplementation of 25(OH)D_3_ compared to Vit D_3_. The increased concentration of 25(OH)D_3_ was associated with the positive modulation of parameters related to the humoral immune systems. In contrast to Vit D_3_, dietary supplementation of 25(OH)D_3_ led to a significant increase in the serum concentration of IgG and lysozyme. So, unlike Vit D_3_, the dietary supplementation of 25(OH)D_3_ significantly improved humoral immunity and strengthened the immune response of the piglets without compromising their health.

Thus, unlike Vit D_3_, the dietary intake of 25(OH)D_3_ significantly improves immune status of the body. In particular, the improvement of humoral immunity is reflected in the increased concentration of serum immunoglobulins and phagocytic capacity of the macrophages in piglets, which allows the animals to respond more effectively to potential health challenges. 25(OH)D_3_ also positively affects the serum concentration of inflammatory cytokines to maintain immune homoeostasis. Moreover, 25(OH)D_3_ is superior over Vit D_3_ in modulating the systemic and mucosal antimicrobial responses, suggested by increased numbers of leucocytes and their survival and phagocytic ability in blood and bronchoalveolar compartments. Compared to Vit D_3_, the significant expression of genes related to fatty acid metabolism due to dietary intake of 25(OH)D_3_ is also noteworthy. Fatty acids positively affect immune cell functions through a variety of complex mechanisms by improving phagocytosis, T-cell signalling and antigen presentation capability^(50)^. In contrast to Vit D_3_, the role of 25(OH)D_3_ in improving gut immunity is also noteworthy. Dietary supplementation of 25(OH)D_3_ alters the gut microbiota and thus might promote specific metabolic processes, which plays a crucial role in maintaining intestinal health^(51)^.

## Comparative performance of vitamin D_3_ and 25(OH)D_3_ on bone development

Vit D regulates bone and mineral metabolism. This section reviews the available pig studies highlighting the efficacy of Vit D_3_ and 25(OH)D_3_ on mineral status and bone development (Table 3).

**Table 3.** Overview of the comparative studies between vitamin D_3_ (Vit D_3_) and 25(OH)D_3_, demonstrating the study designs, dietary doses, underlying conditions and significant outputs of the experiments corresponding to skeletal development. Only the dietary doses (control and treatment) and the conditions that led to noticeable outcomes are listed

Authors	*n*	DTP† (days)	Doses of vitamin D (µg/kg)		Conditions	Outcomes linked to bone development*	
			Vit D_3_ ‡	25(OH)D_3_		Vit D_3_ > 25(OH)D_3_	25(OH)D_3_ > Vit D_3_
Zhang *et al.*^(10)^	144 piglets	14/28	62·5	50	14 d	–	↑Ca (serum)
					28 d	–	↑Ca, ↑BALP^e^, ↑OC^f^ (serum)
Zhao *et al.*^(31)^	240 sows	–	50	50	6–10 kg	–	–
					10–20 kg	–	↑Ca (serum)
Doherty *et al.*^(59)^	24 boars	66	50	25 (Vit D_3_) + 25	–	↑Faecal Ca^d^	↑Ca digestibility, ↑Ca retention
Weber *et al.*^(19)^	227 sows	–	50	50	IN^b^	↑CrossLaps (plasma)	–
					PI^c^	–	↑CrossLaps (plasma)
Witschi *et al.*^(29)^	39 sows	ET^a^	50	50	–	–	–
Rosenberg *et al.*^(62)^	48 piglets	42	50	50	–	–	–

^a^ET, explained on text; ^b^IN, insemination; ^c^PI, post-insemination; ^d^Ca, calcium; ^e^BALP, bone-specific alkaline phosphatase; ^f^OC, osteocalcin.

* Asterisk indicates significant outcomes (*P* < 0·05).

† DTP, dietary treatment period.

‡ 1 µg = 40 μg Vit D_3_.

Zhang *et al.*^(10)^ compared the efficacy of Vit D_3_ and 25(OH)D_3_ in improving serum mineral status. In contrast to Vit D_3_ (NC), the dietary inclusion of 25(OH)D_3_ (NC + 25D) significantly normalised the serum levels of Ca, bone-specific alkaline phosphatase and osteocalcin (OC) in low Ca-P fed pigs depending on the duration of the dietary supplementation. The increased serum concentration of Ca indicates the compensatory efforts of 25(OH)D_3_ to maintain Ca-P homoeostasis in pigs fed with low Ca-P diets. Serum level of bone-specific alkaline phosphatase (synthesised by osteoblasts) positively correlates with bone formation^(52)^. OC is also considered as a serum marker of osteoblastic bone formation which acts in the bone matrix to regulate bone mineralisation via resorption^(53)^. Unlike OC, the supplementation of piglets with both forms of Vit D exhibited a similar impact on the serum status of bone resorption markers like tartrate-resistant acid phosphatase (TRAP) and pyridinoline (PYD). TRAPs perform bone resorption by catalysing the hydrolysis of various phosphates and anhydrides in an acidic environment,^(54)^ and PYD is synthesised by the reaction at the side chain of the collagen residues during trimerisation in skeletal development^(55)^. Overall, 25(OH)D_3_-enriched diets proved to be more effective in improving bone integrity in low Ca-P-fed pigs compared with Vit D_3_, as indicated by increased serum bone formation biomarkers to balance bone mineralisation and its homoeostasis.

Similar to the study of Zhang *et al.*^(10)^, Zhao *et al.*^(31)^ also demonstrated the superiority of dietary 25(OH)D_3_ over Vit D_3_ in restoring bone mineral status in pigs supplemented with low Ca-P. Unlike Vit D_3_ (NC), the serum level of Ca increased significantly in piglets (10–20 kg) after dietary ingestion of 25(OH)D_3_ (HyD). Serum Ca needs to be regulated in narrow physiological ranges and serves as an important marker of bone turnover and osteoblast functions^(56,57)^. Its elevation in piglets fed low Ca-P indicates the body’s compensatory effort to balance the level of Ca and P to achieve optimal mineralisation. However, no major dietary effect on the serum status of Ca was observed in 6–10 kg piglets. Dietary supplementation of Vit D had also no discernible effect on the serum levels of P. This could be due to the body’s ability to maintain serum P levels in pigs fed low P^(3,58)^. Thus, the supplementation of dietary 25(OH)D_3_ led to a significant impact on increasing the serum Ca to improve the mineral homeostasis in pigs receiving low Ca-P diet.

Doherty *et al.*^(59)^ performed a similar experiment, but with a different dosage of 25(OH)D_3_ compared with the aforementioned studies. For the mineral balance study, twenty-four finishing boars (13 weeks of age) with an initial live weight of 42 kg were used and fed the following diets containing low P (T1, 50 µg/kg Vit D_3_), T1 + Phytase (T2, 50 µg/kg Vit D_3_ + 750 μg/kg phytase), T1 + 25(OH)D_3_ (T3, 25 µg/kg Vit D_3_ + 25 µg/kg 25(OH)D_3_) or T1 + Phytase + 25(OH)D_3_ (T4, 25 µg/kg Vit D_3_ + 750 μg/kg Phytase + 25 µg/kg 25(OH)D_3_) for 66 d. Accordingly, in contrast to Vit D_3_ (T1), dietary inclusion of 25(OH)D_3_ (T3) significantly improved Ca digestibility, and retention in low Ca-P fed pigs, suggesting the same remedial endeavour as reported in the above studies to balance mineral homeostasis. Ca digestibility and P retention are closely associated with bone mineralisation and growth performance^(60)^. However, the dietary intervention did not affect P metabolism, serum mineral (Ca, P) status, bone ash and bone strength.

Weber *et al.*^(19)^ compared the effectiveness of Vit D_3_ and 25(OH)D_3_ to determine the plasma mineral status following the supplementation of sows with basal diets containing Vit D_3_ and 25(OH)D_3_. Dietary intake of Vit D_3_ and 25(OH)D_3_ did not consistently affect the plasma status of Ca, P and OC associated with skeletal development and resorption throughout the reproduction cycle. However, the concentration of CrossLaps (*β*-CTx) increased significantly before parturition in both treatments and was the highest at the end of lactation. This observation suggests the mobilisation of Ca from the sow’s skeleton to the developing foetuses and piglets for their optimum bone development^(61)^. Thus, in healthy pigs, Vit D_3_ and 25(OH)D_3_ resulted in similar impacts on the concentrations of bone mineralisation markers.

In the study of Witschi *et al.*^(29)^, thirty-nine sows (thirteen in each treatment) were assigned to Vit D_3_ and 25(OH)D_3_ supplementation from the day of mating until day 21 of lactation. Rosenberg *et al.*^(62)^ used six-week-old piglets (*n* 40) supplemented with Vit D_3_ (control, 50 µg/kg) and 25(OH)D_3_ (50 or 250 or 500 µg/kg) for 42 d. Witschi *et al.* and Rosenberg *et al.* reported no significant difference in ash%, weight, length, mineral content and mineral density of bone in response to the dietary intakes of Vit D_3_ and 25(OH)D_3_. However, low dietary intake of Vit D_3_ (200 μg) was associated with reduced bone-breaking strength, cortical bone mineral content and density at the tibial midshaft of the piglets^(29)^. Thus, both forms of Vit D implied a similar effect on bone formation in healthy pigs.

In summary, 25(OH)D_3_ outperforms Vit D_3_ to improve the serum mineral status associated with bone mineralisation in pigs fed low Ca-P diet. Unlike Vit D_3_, the serum concentrations of Ca, bone-specific alkaline phosphatase and OC increase significantly in pigs receiving basal diets containing 25(OH)D_3_ and low Ca-P. Therefore, the upregulation of serum markers associated with bone turnover indicates a noticeable positive influence of 25(OH)D_3_ to balance mineral homeostasis in pigs experiencing Ca-P deficiency. The comparative efficacy of Vit D_3_ and 25(OH)D_3_ on mineralisation in healthy pigs appears to be similar.

## Implications for further research

Vitamin D metabolism is attributed to endogenous adaptive mechanisms for maintaining mineral homeostasis, which is critical for animal health. In particular, potential long-term consequences due to nutritional strategies in the early life stages need to be addressed as summarised in the concept of nutritional programming, thereby inducing resource-efficient phenotypes. However, evaluation must also consider a range of effects in peripheral tissues to account for unintended metabolic effects (Fig. 1). With regard to alternative housing conditions of pigs, such as exposure to natural sunlight, dietary Vit D requirements and the physiological synergy with endogenous syntheses need to be investigated. Identifying the complex genetic architecture of the Vit D system is key to improving mineral efficiency and animal health aspects as a basis for developing new breeding criteria.

### Conclusions

In sows, dietary supplementation of Vit D_3_ and 25(OH)D_3_ results in similar reproductive outcomes. The growth performance of piglets and sows fed 25(OH)D_3_ is significantly better than those fed Vit D_3_. The superiority of 25(OH)D_3_ over Vit D_3_ is evident in enhancing innate and humoural immunity. Improved bone mineralisation in pigs supplemented with 25(OH)D_3_ on diets low in Ca and phosphorus provides an option to balance animal welfare and resource efficiency. Thus, 25(OH)D_3_ is a promising and potential alternative to Vit D_3_ for promoting growth, immunity and bone development in the pigs.

## Supplementary material

For supplementary material accompanying this paper visit https://doi.org/10.1017/S0007114523000442. click here to view supplementary material
