# Efficacy of dietary vitamin D_3_ and 25(OH)D_3_ on reproductive capacities, growth performance, immunity, and bone development in pigs – CORRIGENDUM

**DOI:** 10.1017/S000711452300079X

**Published:** 2023-11-28

**Authors:** Maruf Hasan, Michael Oster, Henry Reyer, Klaus Wimmers, Dagmar-Christiane Fischer

**Details of correction:** correct footnotes to tables 1, 2 and 3; please correct each one

**Existing text:** in the footnotes of tables 1,2 and 3:

1 µg = 40 **µg** Vit D3

**Corrected text should read:**

1 µg = 40 **IU** Vit D3

