# Supplementary material for: Efficacy of dietary vitamin D3 and 25(OH)D3 on reproductive capacities, growth performance, immunity and bone development in pigs
Source: Br J Nutr. 2023 Feb 27;130(8):1298–307. doi: 10.1017/S0007114523000442 (PMC10511684; doi:10.1017/S0007114523000442)
Supplement: Supplementary file 1 [file S0007114523000442sup.zip › S0007114523000442sup001.docx]

**Identification of studies via databases and registers**

Records identified from

Databases*

(n = 695)

**Identification**

Records screened

(n = 695)

Records excluded lacking comparision of VitD3 and 25(OH)D3 (n = 655)

**Screening**

Reports excluded:

No dietary composition (n = 3)

Duplicate (n = 2)

Reports assessed for eligibility after proper evaluation

(n = 40)

Studies included in review (n = 35)

Reports of included studies (n = 35)

**Included**

**Source of flow diagram**: Prisma 2020 statement

*Web of Science, PubMed; the following search terms were used:

**Web of Science**

- (Vitamin D) AND (Growth OR Immunity) AND (Pig* OR Sow* OR Piglet* OR Boar* OR Swine*) (Topic)
  - Hits: 454
  - Searched on: 23.05.2022

**PubMed**

- (Vitamin D) AND (Growth OR Immunity) AND (Pig OR Sow OR Piglet OR Boar OR Swine)
  - Hits: 169
  - Searched on: 01.06.2022
- ("Vitamin D"[Mesh]) AND ( "Swine/growth AND development"[Mesh] OR "Swine/immunology"[Mesh] )
  - Hits: 26
  - Searched on: 02.06.2022
- (( "Swine/growth and development"[Mesh] OR "Swine/immunology"[Mesh] )) AND "Cholecalciferol"[Mesh]
  - Hits: 18
  - Searched on: 02.06.2022
- ("Vitamin D"[Mesh]) AND "Swine/growth AND development"[Mesh]
  - Hits: 23
  - Searched on 02.06.2022
- ("Vitamin D"[Mesh]) AND "Swine/immunology"[Mesh]
  - Hits: 5
  - Searched on: 02.06.2022
